# Neuropsychiatric Presentations in an Individual With a Pineal Cyst During Perinatal Period: A Case Report

**DOI:** 10.1192/bjo.2026.11795

**Published:** 2026-06-30

**Authors:** Pyone Aye Kyi, Hnin Thiri Wai, Daniela Hooper

**Affiliations:** Essex, Colchester, United Kingdom

## Abstract

**Aims:**

Pineal cysts are frequently identified as incidental findings on neuroimaging and are generally considered benign. However, the pineal gland plays a central role in melatonin secretion and circadian rhythm regulation, processes closely linked to mood stability, sleep, and affective vulnerability. Neuropsychiatric presentations occurring alongside pineal cysts, particularly during hormonally and circadian-sensitive periods such as the perinatal phase, remain poorly understood.

**Methods:**

This case report describes a 37-year-old woman, employed as a nurse, who experienced recurrent affective episodes over a ten-year period. A comprehensive psychiatric assessment was conducted. Neuroimaging findings were reviewed alongside neurological and psychiatric management. The patient’s clinical course across four episodes was documented, with particular focus on the most recent episode occurring in the postpartum period and managed using a multidisciplinary team approach.

**Results:**

The patient experienced four episodes characterised by manic symptoms such as elevated and labile mood, marked anxiety, reduced need for sleep, and transient psychotic features. No depressive episodes were reported. The most recent episode occurred approximately four months postpartum, following a stressful Caesarean section and significant sleep disruption related to infant care. There was a family history of depression but no history of neurological symptoms. Magnetic resonance imaging during the first episode identified a pineal cyst measuring 8 mm with a well-defined rim which remained stable on subsequent imaging and demonstrated no mass effect on adjacent structures. Neurological assessment concluded that no surgical or disease-specific intervention was indicated. Psychiatric management consisted of low-dose olanzapine, short-term sedative medication, and supportive interventions targeting sleep restoration, resulting in good symptomatic recovery after each episode. Prominent sleep–wake cycle disruption was a consistent feature across all presentations. Melatonin was not prescribed, though its potential role in sleep regulation and relapse prevention was considered.

**Conclusion:**

This case illustrates the diagnostic complexity that arises when recurrent affective symptoms coexist with incidental neuroimaging findings. Although a causal relationship between pineal cysts and affective episodes cannot be established, the pineal gland’s role in circadian regulation suggests a possible indirect neuroendocrine contribution, particularly during the perinatal period. The case highlights the importance of integrating neurobiological, circadian, and phenomenological perspectives in psychiatric assessment and management.

